# The loss of STAT3 in mature osteoclasts has detrimental effects on bone structure

**DOI:** 10.1371/journal.pone.0236891

**Published:** 2020-07-30

**Authors:** Rebecca K. Davidson, Evan R. Himes, Shinya Takigawa, Andy Chen, M. Ryne Horn, Tomas Meijome, Joseph M. Wallace, Melissa A. Kacena, Hiroki Yokota, Andrew V. Nguyen, Jiliang Li

**Affiliations:** 1 Department of Biology, Indiana University Purdue University Indianapolis, Indianapolis, Indiana, United States of America; 2 Department of Biomedical Engineering, Indiana University Purdue University Indianapolis, Indianapolis, Indiana, United States of America; 3 Department of Orthopaedic Surgery, Indiana University School of Medicine, Indianapolis, Indiana, United States of America; 4 Department of Biological Sciences and Geology, the City University of New York-Queensborough Community College, Bayside, New York, United States of America; Charles P. Darby Children's Research Institute, 173 Ashley Avenue, Charleston, SC 29425, USA, UNITED STATES

## Abstract

Signal Transducer and Activator of Transcription 3 (STAT3) has recently been shown to be involved in bone development and has been implicated in bone diseases, such as Job’s Syndrome. Bone growth and changes have been known for many years to differ between sexes with male bones tending to have higher bone mass than female bones and older females tending to lose bone mass at faster rates than older males. Previous studies using conditional knock mice with *Stat3* specifically deleted from the osteoblasts showed both sexes exhibited decreased bone mineral density (BMD) and strength. Using the Cre-Lox system with Cathepsin K promotor driving Cre to target the deletion of the *Stat3* gene in mature osteoclasts (STAT3-cKO mice), we observed that 8-week old STAT3-cKO female femurs exhibited significantly lower BMD and bone mineral content (BMC) compared to littermate control (CN) females. There were no differences in BMD and BMC observed between male knock-out and male CN femurs. However, micro-computed tomography (μCT) analysis showed that both male and female STAT3-cKO mice had significant decreases in bone volume/tissue volume (BV/TV). Bone histomorphometry analysis of the distal femur, further revealed a decrease in bone formation rate and mineralizing surface/bone surface (MS/BS) with a significant decrease in osteoclast surface in female, but not male, STAT3-cKO mice. Profiling gene expression in an osteoclastic cell line with a knockdown of STAT3 showed an upregulation of a number of genes that are directly regulated by estrogen receptors. These data collectively suggest that regulation of STAT3 differs in male and female osteoclasts and that inactivation of STAT3 in osteoclasts affects bone turnover more in females than males, demonstrating the complicated nature of STAT3 signaling pathways in osteoclastogenesis. Drugs targeting the STAT3 pathway may be used for treatment of diseases such as Job’s Syndrome and osteoporosis.

## Introduction

Previous studies have shown that bones develop differently between the sexes. From birth to death, bone size and mass are observed to be greater in males than in females [1, 2]. Studies using growth hormone receptor knock-out mice have demonstrated that bone mass acquisition is regulated by sex hormones, with male androgens stimulating bone size and female estrogens limiting bone size [3, 4]. Regulation of bone development is mediated in part by the interaction between osteoblasts and osteoclasts. A critically important signaling pathway between osteoblast-osteoclast interaction is the RANK-RANKL-OPG pathway [5, 6] with osteoblasts secreting RANKL required for osteoclast differentiation [7]. This interaction is further modified by OPG, a decoy receptor, secreted by bone marrow stromal cells to downregulate RANKL stimulation [8]. Upon RANKL binding to RANK on osteoclasts, TNF receptor-associated factor-6 (TRAF6) is activated leading to the recruitment of several transcriptional factors in the nucleus and thereby turning on osteoclast differentiation genes including cFos, NFATc1, and cathepsin K [9, 10]. OPG deficient mice, for example, exhibit severe osteoporosis due to too much RANKL signaling [11], while transgenic mice with a targeted deletion in the RANK, RANKL, or TRAF6 genes develop severe osteopetrosis [5, 12, 13].

Osteoblast regulation of osteoclast function may be further modified by members of the IL-6 family [14]. The IL-6 family of cytokines include IL-6 complexing with its soluble IL-6 receptor (IL-6-sIL6R), IL-11, oncostatin M (OSM), and leukemia inhibiting factor (LIF), all of which bind to the GP130 receptor on osteoblasts [14–16] that leads to phosphorylation of the Janus kinase family of tyrosine kinases (JAK1, JAK2, JAK3, and Tyk2 [17]). JAK kinases then phosphorylate the signal transducers and activators of transcription (STAT) family of transcription factors, which are comprised of seven members: STAT1, STAT2, STAT3, STAT4, STAT5a, STAT5b, and STAT6. Of the three STAT proteins, STAT3, but not STAT1 and STAT5, appears to be the most important intracellular factor regulating RANKL expression in osteoblasts [18, 19].

We and others [20, 21] have previously demonstrated that STAT3 in osteoblasts is required for bone maintenance. The osteoblast-specific STAT3 knock-out mice exhibited low body weight as well as decreases in BMD compared to the littermate controls [20]. In addition, mechanical strength and growth rate were reduced in osteoblast-specific STAT3 knock-out mice [20]. Load-driven bone formation after dynamic loading of the ulnae from osteoblast or osteocyte specific STAT3 knock-out mice was also decreased, while reactive oxygen species (ROS) levels increased, suggesting that STAT3 might regulate mitochondrial activity in osteoblasts [20, 22].

While the functions of STAT3 in osteoblasts and osteocytes are emerging, the roles of STAT3 in osteoclasts have not been clearly defined. A recent study in which STAT3 in osteoclasts was deleted by use of the Cathepsin K promoter driving Cre recombinase demonstrated that mice exhibited an increase in BMD with a decrease in bone resorption [23]. However, mice in which STAT3 was conditionally deleted using TIE2 promoter driving Cre in early stages of osteoclast development exhibited osteoporosis with significantly higher osteoclast numbers [24]. These studies highlight the contradictory roles of STAT3 in bone homeostasis and thus the need for further examination.

Clinically, STAT3 has been implicated in the autosomal-dominant form of Hyperimmunoglobulin E Syndrome (HIES), also known as Job’s syndrome [25, 26]. HIES patients have an increase in IgE concentration of up to 10 times the expected serum levels [27]. HIES patients exhibit increased numbers of infections as well as skeletal abnormalities including osteoporosis with higher bone resorption, leading to an increase in fracture risk and scoliosis [28, 29]. Although Job’s syndrome is found in both males and females, the effect of loss of STAT3 in males and females has not been examined extensively.

The purpose of this study was to examine the role of STAT3 in mature osteoclasts during skeletal development and to further examine the regulation of STAT3 in osteoclast differentiation *in vitro*. We determined the function of STAT3 in osteoclasts by using the Cre-lox system with the Capthepsin K promoter driving Cre recombinase as a regulator of osteoclast function in bone development. Interestingly, STAT3 regulation of bone resorption appears to be differently regulated between sexes. Furthermore, microarray analysis of RAW264.7 cells with STAT3 miRNA has identified a number of differentially expressed genes that are potential targets of STAT3 regulation, including some known to be regulated by female hormones.

## Materials and methods

### Animals

Indiana University Purdue University Indianapolis School of Science IACUC committee has approved the study protocol. Mice were anesthetized or euthanized with inhalation of isoflurane or carbon dioxide. Eight weeks old C57BL/6 female and male osteoclast-specific STAT3 knock-out mice were created using the Cre-loxP system. Mice expressing Cre recombinase driven by a Cathepsin K (CTSK) promoter [30] were bred with homozygous C57BL/6 mice with floxed STAT3 mice containing two LoxP sequences flanking exons 18–20 of the *Stat3* gene (Jackson Laboratory, Bar Harbar, ME). Conditional knock-out (STAT3-cKO) mice on the C57BL/6 background carried the Cre recombinase transgene and floxed *stat3* region, while the littermate control (CN) C57BL/6 mice carried only the Cre recombinase transgene. Calcein (30mg/kg) and alizarin (50mg/kg) were injected intraperitoneally 7 days and 2 days before euthanasia, respectively. The right femurs from the experimental mice were fixed in 10% formalin overnight and then were transferred into 70% ethanol for bone histomorphometry. The left femurs from the experimental mice were frozen in saline-soaked gauze at -20°C for bone mineral measurement, micro-CT scanning, and biomechanical testing.

### Bone mineral density and bone (BMD) and bone mineral content (BMC) analysis

Left femurs from 8 week old STAT3-cKO and CN C57BL/6 mice underwent ex vivo dual-energy X-ray absorptiometry (DEXA) scanning (Lunar PIXImus, Madison, WI) to calculate BMD (g/cm^2^) and BMC (g).

### Micro-CT (μCT) analysis

Left femurs from 8 week old STAT3-cKO and CN C57BL/6 mice were also scanned using a nominal voxel size of 10 μm (Skyscan 1172, Bruker, Belgium). Scans were performed using a 0.7-degree angle increment, two frames averaged, through a 0.5 mmAL filter (V = 50 kV). The raw images were reconstructed using NRecon software. All data were analyzed using the CTan program and 3D models were constructed using the CTvol program. All volumes of interest were analyzed at a section of the proximal femur beginning 0.5 mm proximally to the growth plate and continuing 1.0 mm proximally.

For the femoral cortical bone analysis, a 1-mm region of interest was selected at the midshaft of the bone. The cortical shaft was analyzed (Matlab, MathWroks, Inc. Natick, MA) to determine the total cross-sectional area (Tt.Ar), cortical area (Ct.Ar), and marrow area (Ma.Ar), cortical thickness (Ct.Th), cortical area fraction (Ct.Ar/Tt.Ar) and principal moments of inertia (I_max_ and I_min_).

### Bone histomorphometry

Right femurs from 8 week old STAT3-cKO and CN C57BL/6 mice were dehydrated in graded alcohol and methyl methacrylate, then embedded in methyl methacrylate. From the distal femurs, 5-μm-thick sections were cut using a microtome (Leica, Germany). For each set of bone sections from the femurs, two unstained sections were mounted on microscope slides, while other sections were stained with tartrate-resistant acid phosphatase (TRAP) to identify active osteoclasts. Plastic embedded sections of the right femur were analyzed with an Olympus BX53 light/fluorescent microscope and Olympus DP72 camera interfaced with Osteomeasure™ software version 1.01 (OsteoMetrics Inc, Decatur GA). Images were analyzed at 200x magnification. Parameters measured were as described by Parfitt et al. [31]. Mice lacking one of the fluorescent labels were given a mineral appositional rate of 0.1μm/day. This avoided a MAR of zero and allowed for calculation of bone formation rates [32]. The primary data were collected from the metaphyseal area, at a 0.5 mm proximal to the growth plate and 0.5 mm away from the intracortical surface. Tissue area (T.Ar), trabecular bone area (tB.Ar), trabecular perimeter (tB.Pm), single label perimeter (sL.Pm), double label perimeter (dL.Pm), inter-label thickness (Ir.L.Ar), osteoclast surface (Oc.S), and osteoclast number (N.Oc) were calculated at that region. The following parameters were calculated: mineralizing surface/bone surface indicated as (MS/BS, %), mineral apposition rate (MAR, μm/day), bone formation rate (BFR/BS, μm^3^/μm^2^ per year) (bone surface referent), percentage of osteoclast surface (Oc.S/BS, %) and osteoclast number per tissue area (Oc.N/T.Ar, #/mm^2^).

### Tartrate Resistant Acid Phosphatase (TRAP) stain

The bone sections from 8 week old STAT3-cKO and CN C57BL/6 mice were deplastified in acetone and rehydrated using ethanols. Slides containing the sections were then incubated in a sodium acetate buffer pH 5.0 containing 0.2 M sodium acetate and 50mM sodium tartrate dibasic dihydrate for 20 minutes. Slides were then transferred to a pH 5.0 sodium acetate buffer containing 0.2 M sodium acetate, 50mM sodium tartrate dibasic dihydrate, 0.5mg/mL napthol AS-MX phosphate, and 1.1 mg/mL fast red TR salt for 1 hour at 37°C. Sections were then counterstained with hematoxylin [33].

### Biomechanical testing of the femoral diaphysis

Bone mechanical integrity was calculated by 3-point bending using femurs from 8 week old STAT3-cKO and CN C57BL/6 mice [34]. Previously frozen femurs were slowly thawed to room temperature before being placed on a loading fixture (Test Resources 100 Series Test Instrument, 25 lb load cell) with a support span set at 6.0 mm (anterior side down). Loads were applied in a posterior-anterior direction at 0.03 mm per second until the failure of the bone. During loading, force and displacement were recorded from which structural strength, stiffness, and work were calculated using a custom MATLAB script. Force/displacement was mapped into stress/strain using μCT data and standard beam-bending equations to estimate tissue-level properties. Because there were no differences in any tissue-level properties, only structural-level properties are reported.

### Primary cell culture

Osteoclast-like cells were generated from murine bone marrow monocytes (BMMs) from two groups of 6 week old, male and female, STAT3-cKO and CN C57BL/6 mice. Experiments using BMMs from each group of mice were completed in triplicate. The resorption activity of cultured osteoclasts was evaluated using a standard resorption assay. Murine BMMs were plated into 6-well culture plates at 2x10^6^ cells/well. Cells were incubated in alpha-MEM containing 10% FBS and 20 ng/ml M-CSF for 2 days. The media was removed and replaced with fresh media containing 20 ng/ml M-CSF and 80 ng/ml RANKL until mature osteoclasts were observed. Mature osteoclasts were detached by trypsinization, washed once, re-plated onto Corning Osteo Assay Surface plates and cultured for an additional 48 hours in media containing 20 ng/ml M-CSF and 80 ng/ml RANKL. The wells were washed, incubated in 6% NaCl for 5 min, and sonicated for 20 s to remove cells. Plates were stained with a solution containing 1% toluidine blue and 1% sodium borate for one minute, washed with water and air-dried. Resorbed surface area (resorption area) was quantified using the ImagePro7.0 on a Leica DMI4000 with a 10X objective and normalized by the total measurement area. The percentage of resorption area was further normalized by TRAP+ osteoclast number.

### Cell culture

RAW 264.7 cells (1 x 10^5^) were seeded in six-well plate in 5 mL of antibiotic-free 10% FCS MEM-alpha medium. The plates were incubated 24 hours before the addition of siRNA Stat3 Silencer (ThermoFisher Scientific). Twenty-five nM of Select siRNA was used to knock down *Stat3* gene expression and New NC2 Custom Select siRNA was used as a negative control. Lipofectamine RNAiMax was used to transfect the cells per the manufacturer’s protocol. After transfection, the plates were incubated for 6 or 24 hours and the medium was changed to 5 mL of MEM-alpha media with 10% FBS and 1% penicillin and streptomycin antibiotic. After the incubation period, the cells were stimulated with 50 ng/ml of RANKL (R & D Systems) or PBS control for 24 hours prior to harvesting the cells and were processed for either Western blot or microarray analysis.

### Western blot analysis

Radioimmunoprecipitation assay (RIPA) lysis buffer (Millipore Sigma, Burlington, MA) was prepared by mixing RIPA with protease inhibitor and phosphatase inhibitor (1:100). The medium was aspirated from the wells and the cells were washed 3x with 5 mL PBS. A volume of 300 μl RIPA buffer was added to each well. The cells were scraped off the wells and transferred to 1.5 mL tubes incubated on ice. The cells were sonicated on ice and centrifuged at 4°C for 10 minutes at 13,200 rpm. The supernatant was transferred to separate tubes and placed on ice for PAGE or stored in the -80^o^ freezer. Antibodies used for this study included1^o^ Ab specific to p-STAT3 (Ser727), NFATc1, c-Fos, and Cathepsin K; all were purchased from Santa Cruz Biotechnology (Dallas, Tx); 1^o^ Ab specific to ß-actin Ab was obtained from Sigma-Aldrich, (St Louis, MO).

### Microarray analysis

RAW264.7 cells were cultured as described above. The cells were divided into 4 groups: 1) RAW264.7 cells with non-specific siRNA and not stimulated with RANKL, 2) RAW264.7 cells with Stat3 siRNA and not stimulated with RANKL 3) RAW264.7 cells with non-specific siRNA stimulated with 50 ng/ml of RANKL, and 4) RAW264.7 cells with Stat3 siRNA stimulated with 50 ng/ml of RANKL. RAW264.7 cells with and without siRNA STAT3 were either untreated or treated with RANKL for 24 hours in triplicate samples before RNA extraction, membrane-hybridization, and microarray were performed. Triplicate RNA samples were extracted for 12 GeneChip^TM^ Mouse 2.0ST arrays (ThermoFisher Scientific). Both RANKL groups were compared and genes with significant changes (p < 0.05) and with a ≥ 1.2 fold change were selected for further analysis using real-time PCR (qPCR).

### Real-time PCR analysis

Total RNA was prepared using RLT Plus buffer (Qiagen, Germantown, MD) plus 2-mercaptoethanol per the manufacturer’s protocol. Briefly, cells were scraped thoroughly and transferred to a Qiashredder tube and centrifuged to remove supernatant. RNeasy spin columns were used to purify total RNA (Qiagen, Germantown, MD). The cDNA was prepared using 1000 ng of each RNA sample, random hexamers and MultiScribe Reverse Transcriptase (ThermoFisher Scientific). Real-time PCR was performed in a 20 μl reaction using cDNA’s, 2x Master Mix (Roche Molecular Systems, Branchburg, NJ) in a PCR7500 Fast system (ThermoFisher Scientific) and custom-designed primers (Primer sequences used in Real-time PCR is listed in S1 Table).

### Statistics

Data were reported as the mean ± standard deviation and analyzed using the Statistical package JMP (Version 14, SAS Institute Inc.). Data for animal experiments were tested using two-way ANOVA with STAT3 cKO and sex as main effects followed by a Student’s t-test post hoc test for comparing the littermate control and STAT3-cKO parameters as two groups for each sex. Microarray and RT-qPCR data were analyzed between the groups with and without Stat3 siRNA under either vehicle or RANKL treatment by a Student’s t-test. Statistical significance was assumed for p < 0.05.

## Results

### STAT3 function in osteoclasts and bone mass

To examine the function of the STAT3 specifically in mature osteoclasts, we analyzed femurs isolated from 8 week old STAT3 -cKO female and male mice and their littermate controls by measuring BMD, BMC and bone geometry (Table 1). No significant changes in body weight were identified between age- and sex-matched STAT3-cKO and CN mice. Similarly, no major differences in 8-week femur lengths were observed between age- and sex-matched STAT3-cKO and CN mice. However, femurs at 8-weeks of age, showed a significant decrease in BMD in STAT3-cKO females, decreasing by 8.6% (p < 0.05) relative to the control group (Table 1). Likewise, the BMC of the femurs from STAT3-cKO female mice showed a decline of 13.6% (p < 0.05) compared to the control femurs. There was, however, no difference in the BMD nor BMC in femurs of male STAT3-cKO and male control mice.

**Table 1:** Body weight, femoral geometry, mineral content and density, and mechanical properties in 8 week old male and female STAT3-cKO mice and their littermate controls (CN).

	**Females**		**Males**	
	**CN (n = 11)**	**STAT3-cKO (n = 12)**	**CN (n = 9)**	**STAT3-cKO (n = 10)**
**Body weight (g)**	18.01±0.65	18.24±1.49	22.10±0.95	22.93±1.96
**Femur Length (mm)**	14.02±0.53	14.56±0.72	14.62±0.35	15.35±0.73
**Femur BMD (g/cm^2^)**	0.043±0.003	0.039±0.003*	0.046±0.004	0.046±0.007
**Femur BMC (g)**	0.016±0.003	0.014±0.002*	0.019±0.002	0.018±0.004
**Femoral Midshaft Geometry and Mechanical Properties (8 weeks)**				
**Tt.Ar (mm^2^)**	1.53±0.07	1.47±0.10	1.69±0.09	1.78±0.20
**Ct.Ar (mm^2^)**	0.56±0.03	0.53±0.05	0.68±0.05	0.68±0.12
**Ma.Ar (mm^2^)**	0.97±0.05	0.94±0.06	1.01±0.06	1.10±0.10
**Ct.Th (mm)**	0.14±0.01	0.14±0.01	0.17±0.01	0.16±0.02
**Ultimate Force (N)**	10.07±1.3	9.81±2.08	12.41±1.65	14.29±3.09
**Stiffness (N/mm)**	49.9±8.24	46.35±12.24	57.94±9.51	71.81±20.6
**Total Work (mJ)**	11.24±2.67	8.63±2.7*	13.87±4.44	13.23±5.23

BMD: bone mineral density; BMC: bone mineral content.

Tt.Ar: total cross sectional area. Ct.Ar: cortical area. Ma.Ar: marrow area. Ct.Th: Cortical thickness.

*: p < 0.05 v.s. the same sex CN.

### STAT3 is required for bone structure integrity

**Fig 1:**
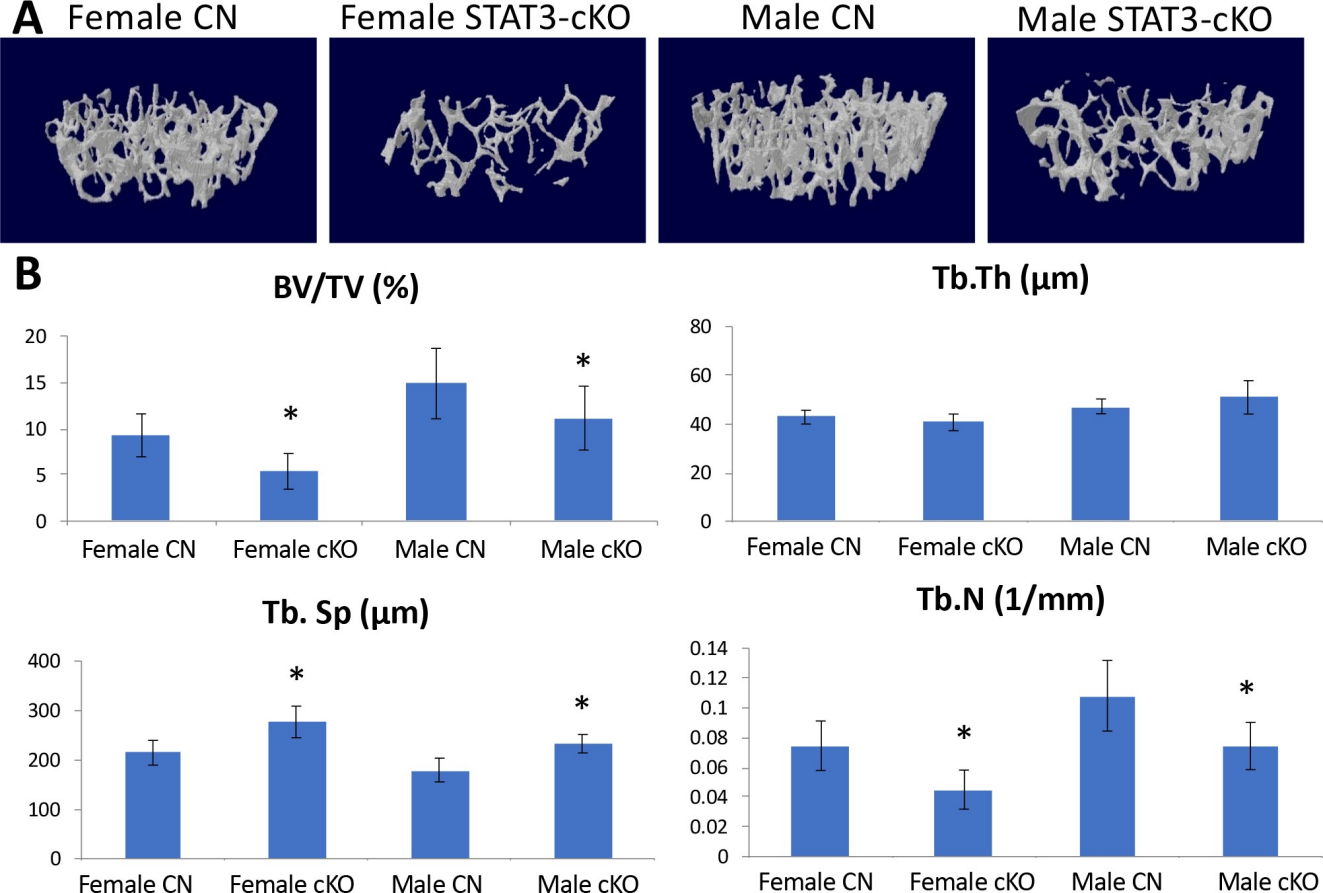
Analysis of the trabecular bone structure of 8 week old femurs from female and male, CN and STAT3-cKO mice. A) Three-dimensional models of a 0.5 mm thick trabecular bone area 1mm proximal from the growth plate. B) Graphs depicting data gathered from the same area. Trabecular number (Tb.N) and bone volume/tissue volume (BV/TV) were significantly decreased in STAT3-cKO males and females compared to age- and sex-matched littermate controls, while trabecular separation (Tb.Sp) was significantly higher in STAT3-cKO female and male mice compared to age- and sex-matched the controls (*: p < 0.05 compared to sex-matched the control, n = 11-14/group).

Femurs from 8 week old male and female STAT3-cKO C57BL/6 mice and their littermate CN were analyzed by μCT. Fig 1A displays representative 3D reconstructions from female and male, control and STAT3-cKO mice. With respect to trabecular bone parameters, the bone volume fraction or bone volume/tissue volume (BV/TV) was significantly decreased by 42% and 25% (p < 0.05) in female and male STAT3-cKO mice, respectively, compared to the controls (Fig 1B). The trabecular number (Tb.N) was also significantly reduced by 40% in female and 31% in male STAT3-cKO mice while trabecular separation (Tb.Sp) increased significantly by 22% and 23% among STAT3-cKO females and males, respectively, compared to the controls (Fig 1B). The decrease in Tb.N was consistent with bigger space between the trabeculae or an increase in Tb.Sp. Of note, no differences in trabecular thickness (Tb.Th) were observed. Collectively, these data suggest that functional STAT3 in osteoclasts is required for skeletal development of trabecular bone in both female and male mice.

### STAT3 function in osteoclastic bone resorption activity

**Fig 2:**
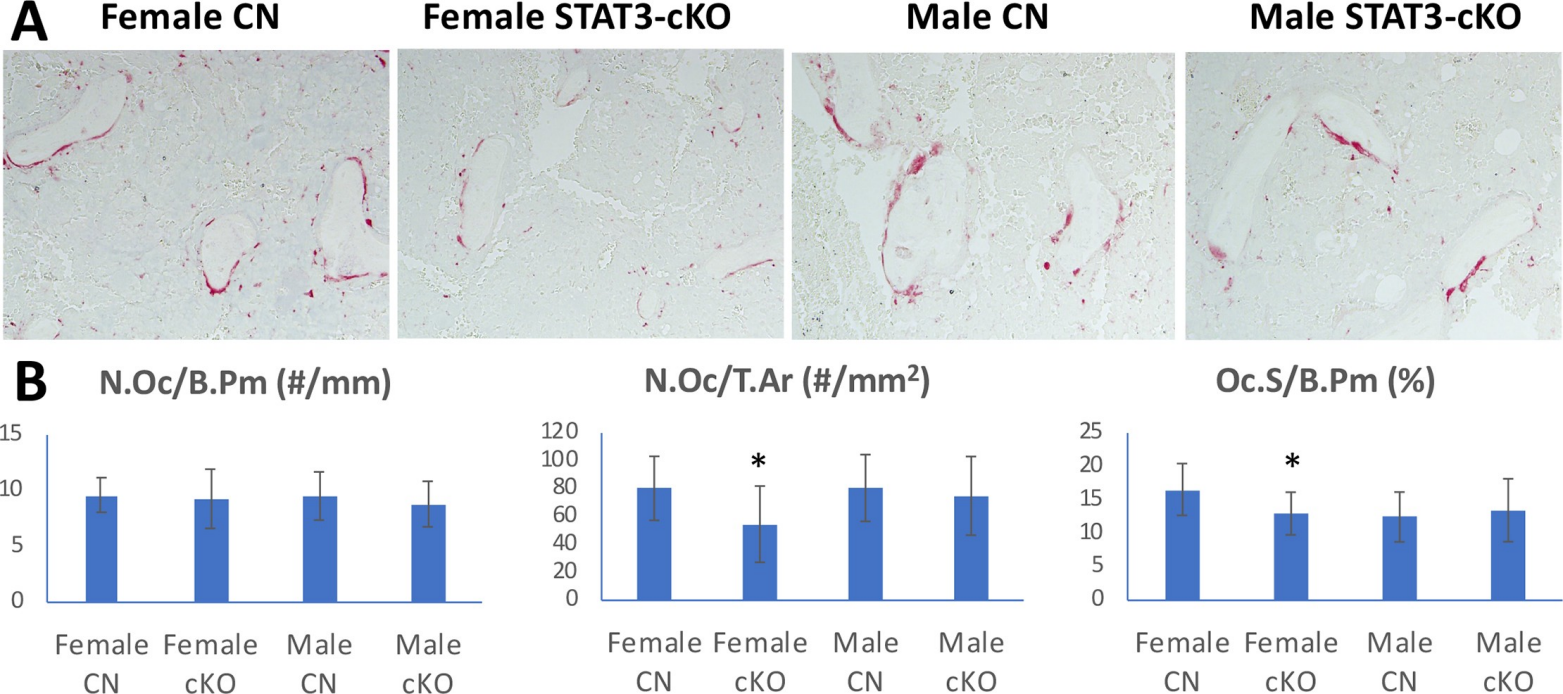
Tartrate Resistant Acid Phosphatase (TRAP) stain of femurs collected from 8 week old female and male control (CN) and STAT3-cKO mice. TRAP+ osteoclasts (red color) of the distal femur (A) and graphical representation of osteoclast number/bone perimeter (N.Oc/B.Pm), osteoclast number/total area (N.Oc/T.Ar) and osteoclast surface/bone perimeter (Oc.S/B.Pm) (B). All data were collected from a region 0.5 mm proximal from the growth plate of the right femur (*: p < 0.05 compared to sex-matched littermate control, n = 11-16/group).

Histochemical staining for the presence of TRAP+ cells (osteoclasts) at the distal femurs showed that N.Oc/B.Pm (osteoclast number/bone perimeter) was not significantly different among 8 week old STAT3-cKO female and male mice and littermate controls (Fig 2A and 2B). But, osteoclast number/tissue area (N.Oc/T.Ar) was significantly decreased in the femurs of female STAT3-cKO vs. control femurs but no changes were observed in male STAT3-cKO femurs vs control femurs (Fig 2A and 2B). Specifically, the osteoclast surface/bone perimeter (Oc.S/B.Pm) was significantly reduced by 36% in STAT3-cKO female vs. control female mice. However, no significant changes were observed between STAT3-cKO male and the control male mice. Thus, the loss of STAT3 in osteoclasts affected trabecular bone volume irrespective of sex, but the changes in osteoclast parameters were only observed in female STAT3-cKO mice.

### The loss of STAT3 in osteoclasts affects bone formation and mineralization in female mice

**Fig 3:**
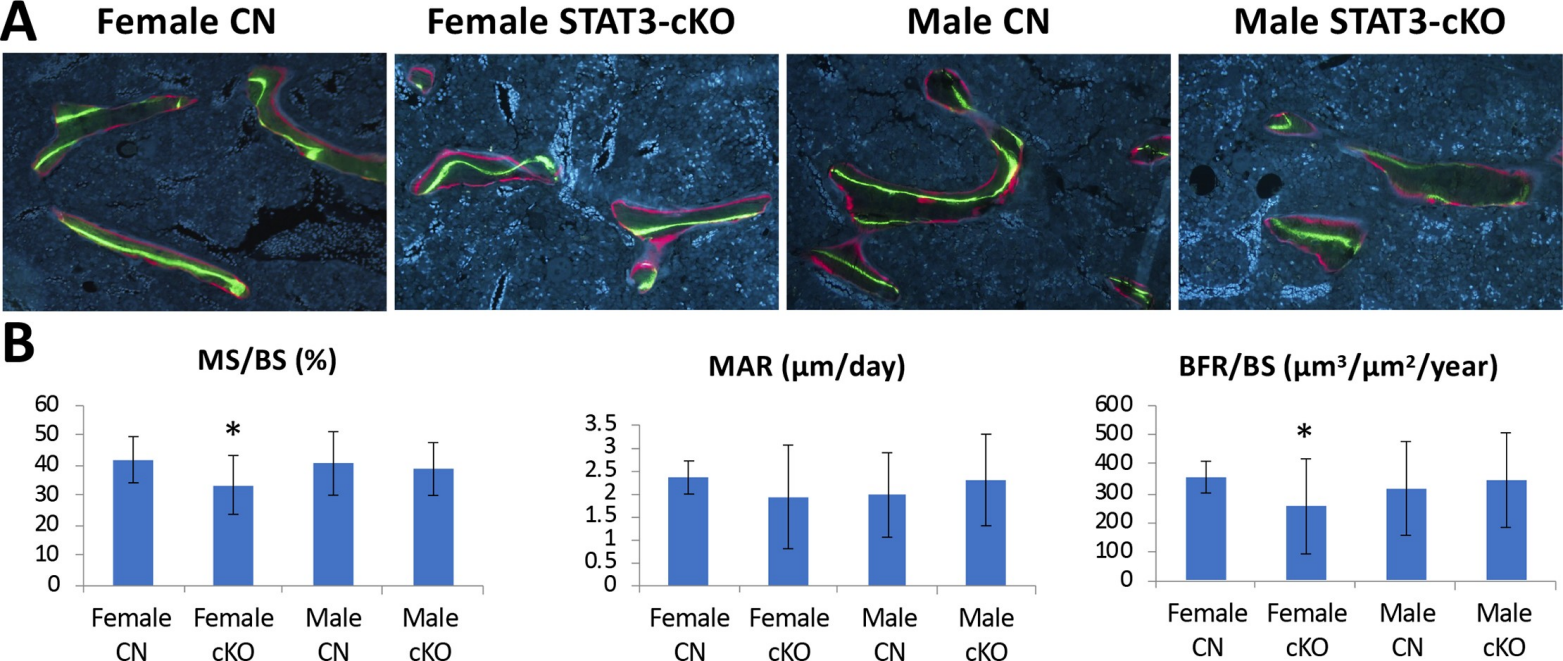
Dynamic histomorphometry analysis of femurs labeled with calcein and alizarin fluorescent dyes. A) Representative figures of distal femurs from 8 weeks old female and male control (CN) and STAT3-cKO mice. B) Data include mineral surface/bone surface (MS/BS), mineral apposition rate (MAR), and bone formation rate/bone surface (BFR/BS). The region 0.5 mm proximal from the growth plate of each was analyzed (* p < 0.05 compared to sex-matched controls, n = 11-17/group).

To determine whether the STAT deficiency in osteoclasts has any effects on bone formation, we used dynamic histomorphometry (Fig 3A). There was a significant, 28% reduction in bone formation rate (BFR/BS) in femurs from female STAT3-cKO mice compared to female control femurs (Fig 3B). No significant changes were observed between femurs from male STAT3-cKO and control mice. The mineralizing surface/bone surface (MS/BS) was also significantly reduced by 20% in femurs from STAT3-cKO females compared to the control females, but remained unchanged in femurs from male mice. With regard to mineral apposition rate (MAR), no significant differences were detected between sex- and age-matched femurs from STAT3-cKO and control mice. Therefore, it appears that targeted deletion of STAT3 in osteoclasts results in reductions in BFR/BS and MS/BS in the femurs from female mice, but not male mice. We further measured osteoblast number, osteoblast surface and osteoid surface as shown in S1 Fig. But, no difference in these parameters were observed, likely owing to the large variations within each group. These data suggest that the lower femur bone mass of STAT3-cKO mice resulted from a decrease in osteoblast recruitment and bone formation rate in femurs of female STAT3-cKO mice.

### Female STAT3-cKO mice have a lower total work

**Fig 4:**
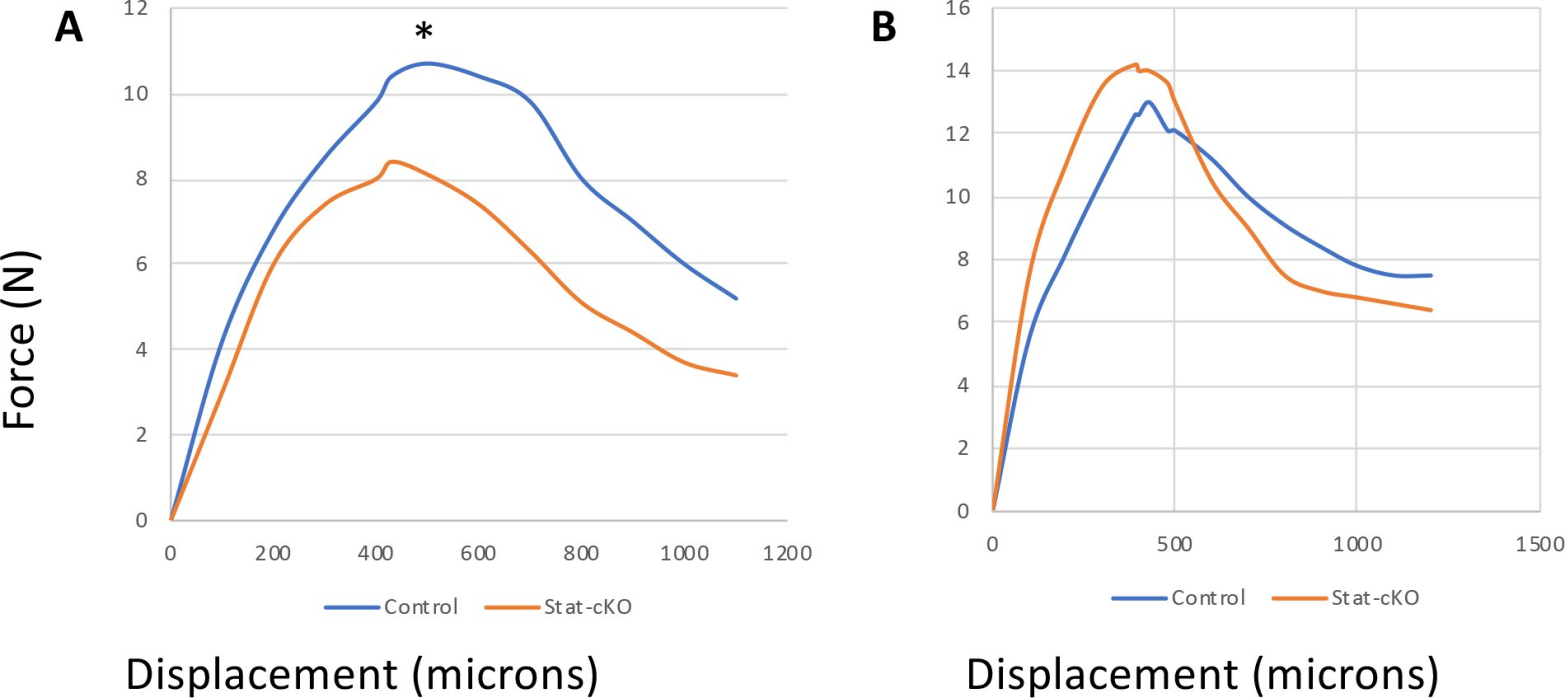
Mechanical testing of female (A) and male (B) STAT3-cKO and control (CN) femurs showing force-displacement curves. Femurs from 8 week old female (A) and male (B) STAT3-cKO and control mice were subjected to 3-point bending to determine femur mechanical properties. Femurs were arranged with their anterior side facing downward, and were loaded with bottom points set 6 mm apart and with the top bending point set in the center. A significant difference was detected between the control female and STAT3-cKO female femurs in total work (A) but not in male control and male STAT3-cKO femurs (B) (*: p<0.05, n = 9-12/group).

To test whether the changes in bone parameters analyzed could affect bone strength, left femurs from 8 week old mice were tested to failure by 3 point bending. The data of the structural mechanical properties and femoral midshaft geometry are shown in Table 1. There were no significant differences in the bone stiffness between femurs from female STAT3-cKO vs. female control mice. Interestingly, male STAT3-cKO femurs showed higher stiffness than male control femurs, but the difference was not significant, perhaps owing to the high variation within the STAT3 KO male femurs. Analysis of the amount of force required to break the bone (ultimate force) showed there were no significant differences detected between sex-matched CN and STAT3-cKO femurs. However, total work, as determined by both force and displacement before fracturing, was significantly decreased in STAT3-cKO female femurs compared to the female control femurs (p < 0.05, Table 1 and Fig 4). No significant differences were observed in male STAT3-cKO femurs vs. male control femurs (Table 1). Of importance, there were no significant differences in geometric measurements in femoral midshaft between sex-matched STAT-cKO and control mice (Tt.Ar, Ct. Ar, and Ma.Ar, Table 1). Collectively, these data suggest that a knockdown of STAT3 in osteoclasts reduces total work in female femurs, but not male femurs.

### Number and activity of bone marrow-derived osteoclasts from STAT3-cKO mice

**Fig 5:**
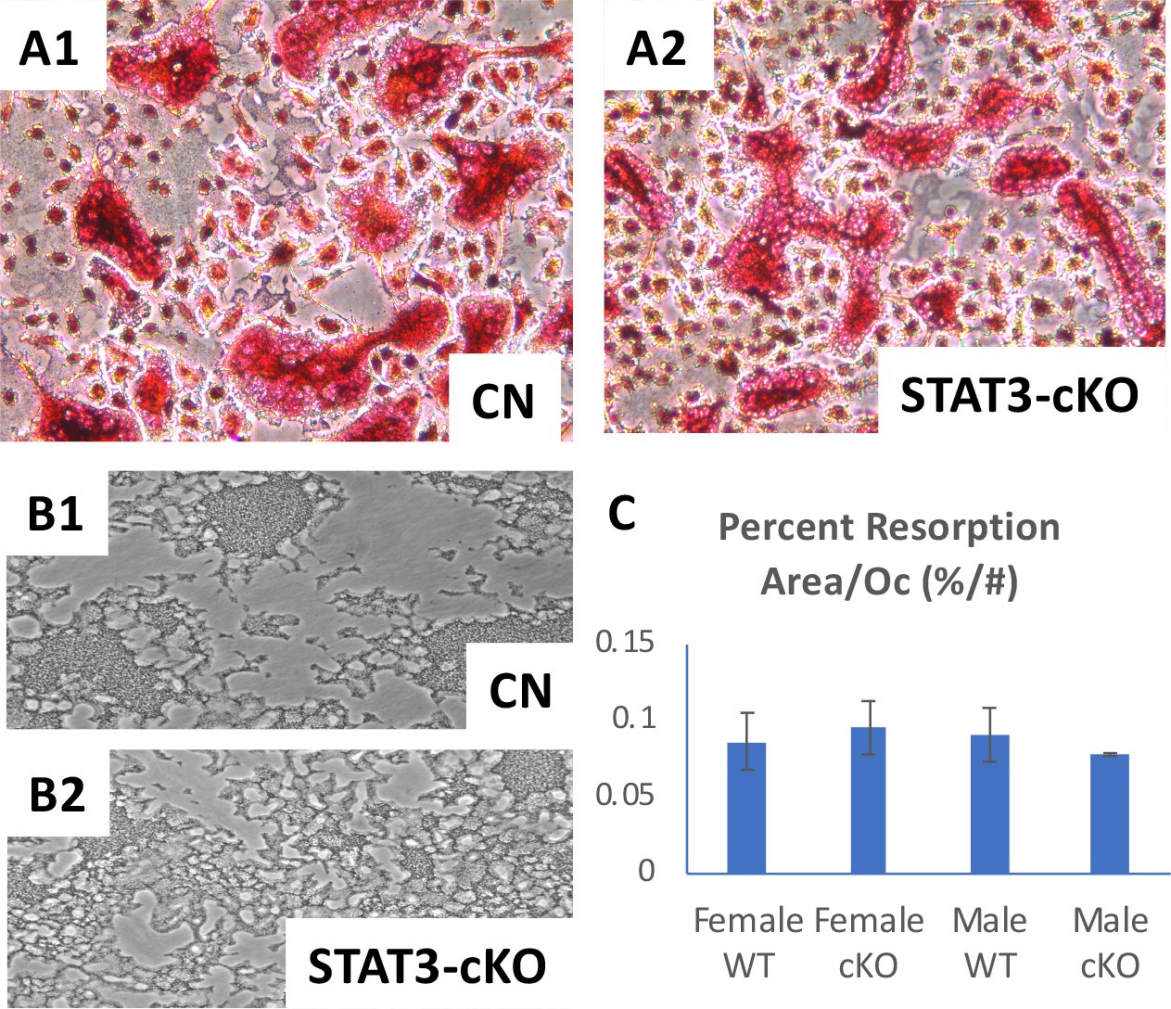
Primary osteoclast-like cells were generated from murine bone marrow monocytes (BMMs) of 6 week old STAT3-cKO and the littermate control (CN) mice and cultured with the addition of M-CSF and RANKL. TRAP stained osteoclasts from CN mice (A1) and STAT3-cKO (A2) (n = 2/group). Resorption assay showed that the resorbed surface appears darker than unresorbed surface of the culture plates using CN (B1) and STAT3-cKO (B2) osteoclasts. C: Quantitation of the percentage resorption area normalized by osteoclast number showed no significant difference between female and male CN and STAT3-cKO osteoclasts.

Since STAT3 deletion from the mature osteoclasts in the STAT3-cKO mice resulted in a difference in bone phenotypes, we further examined the activity of STAT3 deficient osteoclasts *in vitro*. To accomplish this, the whole bone marrow was isolated, and enriched for bone marrow monocytes (BMMs). BMMs from six week old STAT3-cKO male and female mice and littermate controls were then stimulated with M-CSF and RANKL (Fig 5). Analysis of the resorption area did not reveal significant differences in percent resorbed area normalized by osteoclast number between osteoclasts generated from sex-matched STAT3-cKO and control mice (Fig 5C), suggesting the resorption activity per osteoclast did not differ between STAT3-cKO and control osteoclasts *in vitr*o.

### Functional STAT3 regulates osteoclast differentiation via cathepsin K

**Fig 6:**
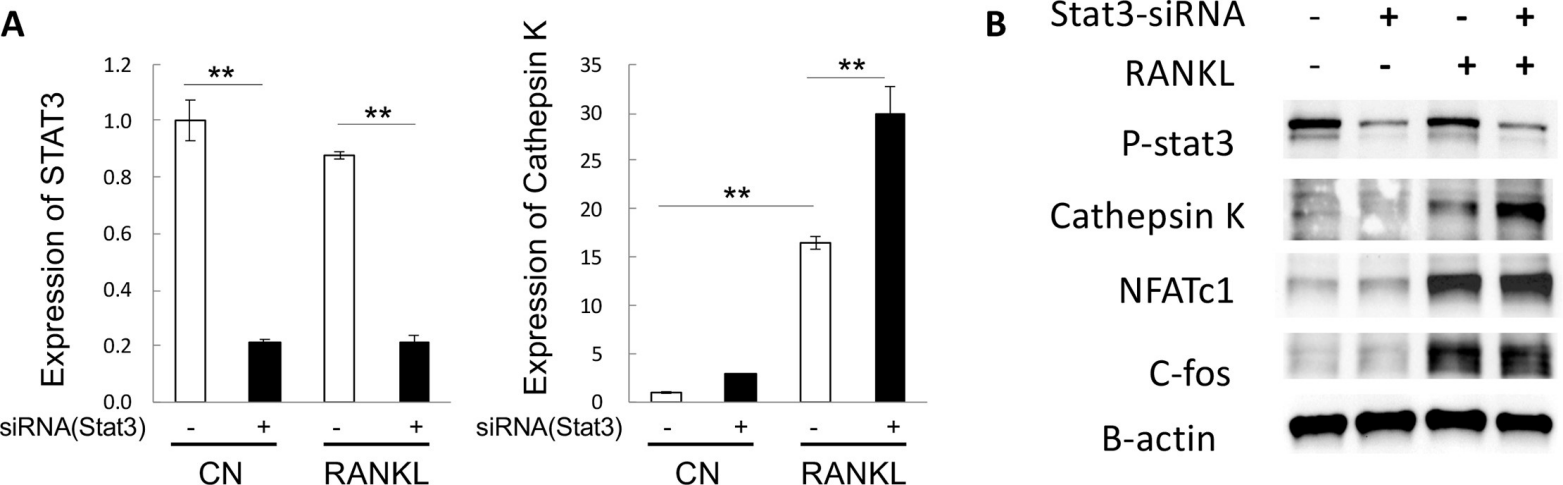
STAT3 is a negative regulator of the expression of cathepsin K. RAW264.7 pre-osteoclastic cells were transfected with Stat3 siRNA and stimulated with RANKL. A) The expression of STAT3 and cathepsin K transcripts was determined by qPCR. *Stat3* mRNA was significantly reduced by Stat3 siRNA. Cathepsin K mRNA was increased by RANKL stimulation by seven fold and further significantly increased by Stat3 siRNA knock down in the RAW264.7 cell by two fold. B) Western blot analysis of proteins expressed in differentiated osteoclasts. After 24 hour siRNA-Stat3 treatment, phosphorylated STAT3 (p-STAT3) was noticeably reduced by Stat3 siRNA. Cathepsin K, NFATc1 and c-fos were markedly increased in response to RANKL treatment. However, only the expression of cathepsin K was further increased in cells treated with both RANKL and Stat3 siRNA knock down. Three independent cell experiments were carried out. ** p < 0.001.

Since STAT3-cKO mice exhibited low BMD and BMC and had some trabecular defects, yet they showed normal osteoclast development (Fig 5), we next wanted to determine whether STAT3 could regulate proteins that are important in osteoclast differentiation and activity. A critical signaling cascade leading to osteoclast differentiation and activity is the stimulation of RANKL, which activates TRAF6 and the Nuclear factor-kappa B (NF-kB) [35]. NF-kB activates c-Fos, which in turn upregulates nuclear factor of activated T-cells 1 (NFATc1) [35]. NFATc1 is the critical transcriptional factor of osteoclast differentiation, which regulates cathepsin K. To determine the function of STAT3 in this pathway, we used Stat3 siRNA to knockdown STAT3 in pre-osteoclastic RAW 264.7 cells and examined the expression of cathepskin K (Fig 6B). SiRNA-STAT3 treatment significantly decreased the mRNA of *Stat3* (Fig 6A). Western blot analyses of the STAT3 siRNA samples showed that p-STAT3 levels were decreased during the 24h of RANKL stimulation (Fig 6B). STAT3 siRNA did not change NFATc1 and c-fos proteins after RANKL stimulation (Fig 6B). The expression of cathepsin K mRNA and protein, however, appears to be strongly induced in samples treated with both STAT3 siRNA and RANKL (Fig 6A and 6B). These data indicate that knockdown of STAT3 stimulated cathepsin K expression.

### Profiling genes regulated by STAT3

**Fig 7:**
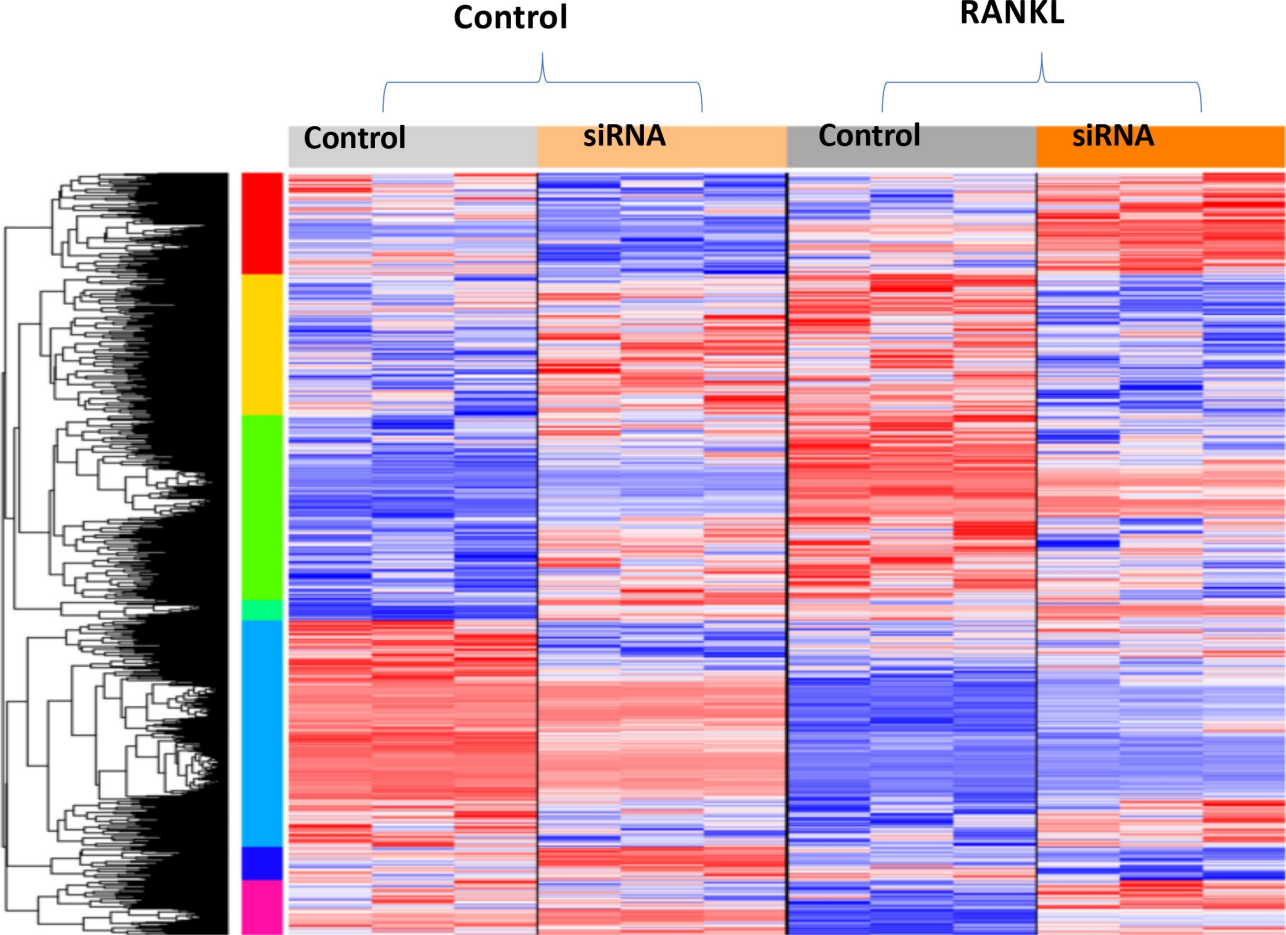
Profiling genes regulated by STAT3. Four groups of RAW264.7 cells: A) 1) unstimulated 2) +siRNA Stat3 unstimulated 3) stimulated with RANKL and 4) +siRNA Stat3 and stimulated with RANKL. Red–upregulated genes, Blue–downregulated genes. The list of genes regulated by RANKL and siRNA STAT3 is presented in S1 Data. The microarray data are also available at GEO (GES152986, https://www.ncbi.nlm.nih.gov/geo/query/acc.cgi?acc=GSE152986).

To further analyze the mechanisms by which STAT3 may function in osteoclasts, a microarray was performed on pre-osteoclastic RAW 264.7 cells. Although the RAW264.7 cell line was derived from male BALB/c mice that was transformed with the Albelson leukemia virus, we chose to use this cell line as it is a widely accepted myeloid cell line and to ensure uniformity within the cell population as opposed to the heterogeneity inherent with working with primary cells such as bone marrow-derived osteoclasts/osteoclast progenitors from control and STAT3-cKO mice. Microarray analysis of 25206 genes revealed that 2018 genes were significantly changed when STAT3 was knocked down in RAW 264.7 cells stimulated with RANKL compared to unstimulated STAT3 knockdown cells (Fig 7, second and fourth column). Microarray data can be found in supplementary S1 Data and have been made available for the scientific community at GEO (Series accession: GES152986, https://www.ncbi.nlm.nih.gov/geo/query/acc.cgi?acc=GSE152986). Of the 296 genes whose fold change is greater than 1.2 in the group with STAT3 siRNA and stimulated by RANKL versus the group with non-specific siRNA and not stimulated by RANKL, 192 genes were significantly upregulated (red) and 104 were significantly downregulated (blue) (p < 0.05). In groups that were stimulated with RANKL with and without siRNA STAT3, 41 differentially expressed genes were identified with a p < 0.05 that had at least a 1.2 fold change (Fig 7, third and fourth column).

### Real-time PCR analysis of genes identified by microarray

**Fig 8:**
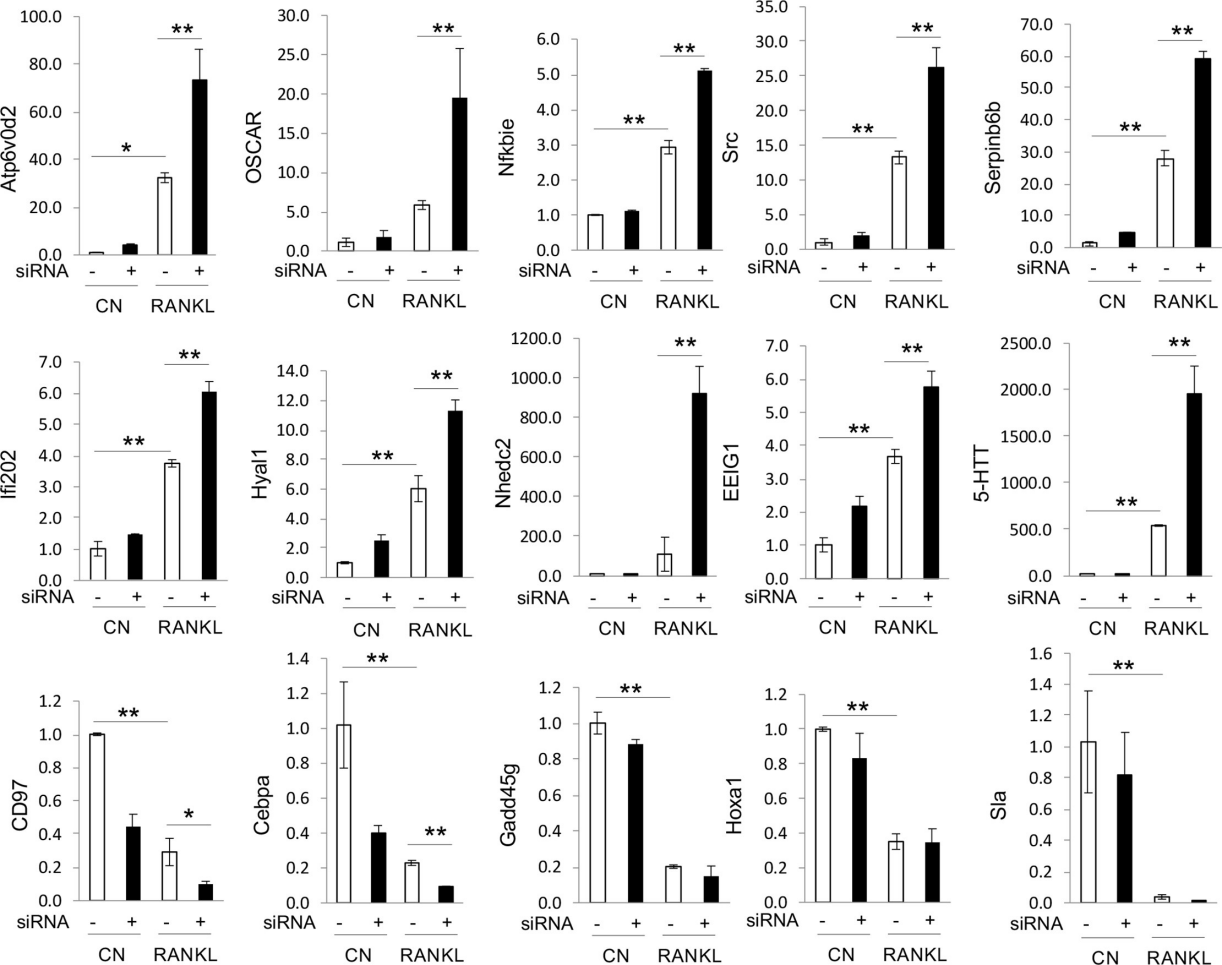
Real-time PCR analysis of genes upregulated or downregulated by STAT3. These genes were identified by microarray analysis. Three independent cell experiments were carried out. * p < 0.01, ** p < 0.001.

Real-time PCR was then used to validate the genes of interest identified as significantly changing by microarray. In brief, another experiment was completed whereby RAW264.7 cells were treated with STAT3 siRNA and stimulated with RANKL for 24h before collecting RNA for gene expression analysis. Ten genes were determined to be upregulated in the absence of STAT3 during RANKL stimulation: Atp6v0d2, OSCAR, Nfkbie, Src, Serpinb6b, lfi202, Hyal1, Nhedc2, EEIG1 and 5-HTT (Fig 8). Five genes (CD97, CEBPA, Gadd45g, HOX-a1, and Sla) were downregulated confirming the pattern seen in the microarray analysis when STAT3 was knocked down during RANKL stimulation (Fig 8).

## Discussion

Our study shows that the loss of STAT3 in mature osteoclasts results in differential bone phenotypes between male and female mice. As some of the observable defects were seen in both sexes due to the loss of STAT3 in osteoclasts, such as the decrease in trabecular bone volume, this may suggest that STAT3 function in osteoclasts is to maintain normal spongy bone development. However, only female femurs with STAT3 knock out in osteoclasts showed decreases in BMD, BMC, lower osteoclast surface, and lower bone formation rate resulting in a lower total work as determined by the three points bending. As these changes were not observed in male femurs with the same knockout, it appears that osteoclast functions are differentially regulated and may be influenced by sex hormones *in vivo*.

Various studies have examined the functions of STAT3 in osteoclasts in bone formation and maintenance, including studies which suggest that STAT3 acting downstream of RANKL mediates osteoclast differentiation [23, 24, 36]. In a mouse study using a conditional knock out of STAT3 with the TIE2-Cre, in which the Cre recombinase is driven by the endothelial-specific promoter/enhancer and is thought to be expressed during an early stage of hematopoietic cell lineage, Zhang et al [24] reported that the loss of STAT3 resulted in mice with an increased osteoclast number and activity with a severe osteoporosis phenotype. These results are consistent with the current study showing lower bone mass despite the discrepancy in osteoclast number, suggesting a slightly different role of STAT3 in osteoclast progenitors and mature osteoclasts.

In a recent study, also with conditional knock-out of STAT3 in osteoclasts using a cathepsin promoter driving Cre recombinase, Yang et al [23] showed that the STAT3 conditional knock-out mice exhibited an increase in bone mass of the 20 weeks old mice in comparison with control mice, the Stat3^LoxP/LoxP^; Cstk-Cre(-) mice. This observation is opposite to our current finding. Of note, the Yang group also report a decrease in osteoclast number in bones with the loss of STAT3 in osteoclasts. In our study, osteoclast number was not different between STAT3-cKO and control mice, but the resorption surface was greater in female STAT3-cKO mice than female control mice. This might be due to the fact that Ctsk-Cre mice used in the Yang et al study were generated by knocking in the Cre recombinase into the Ctsk gene locus [37], whereas our Ctsk-Cre mice were generated by microinjection of Ctsk promoter-Cre into fertilized oocytes [30]. The Yang et al observation of an increase in bone mass could be due to an increase in Cre activity regulated by enhancers around the Ctsk locus, while our Cre recombinase is regulated by the approximately 5Kb promoter of capthepsin K driving Cre gene expression. Furthermore, the STAT3 floxed mice used in the Yang et al study were purchased from Gempharmatech Co. Ltd (Nanjing, Jiangsu, China), which according to the website the mice are kept in the C57BL/6JGpt background while our mice were on the C57BL/6 background. Additionally, Cre negative mice were used as the control in Yang et al study, whereas, we used Cre positive littermate control mice on the C57BL/6 background as the control. Therefore, the discrepancies in bone mass between these two studies might be caused by the differences in genetic backgrounds of mice and the genotypes of the control mice.

In the Yang et al study [23], the downstream targets of STAT3 signaling were examined using cell culture treated with AG490 in an attempt to inhibit STAT3 signaling. A critical factor that one must consider when using JAK2 kinase inhibitors such as AG490 is that it is a very strong inhibitor, but lacks target specificity and thus, it might also downregulate other STAT proteins. Therefore, the conclusion reached from an AG490 study should be taken with a caveat and that STAT3 may not be the only necessary transcriptional factor for osteoclast differentiation. Further, we found AG490 at a dose of 10 uM or higher causes apoptosis of RAW264.7 cells (unpublished observation); thus, we believe that application of Stat3 siRNA to knockdown STAT3 function in cell culture studies is more appropriate. Yang et al also noted that different STAT3 mutants may have different activities on NFATc1. In short, our data suggest that STAT3 acts upstream of NFATc1 and Cathepsin K signaling, and discrepancies in the data observed by our two groups highlight the complicated nature of STAT3 signaling pathways in osteoclastogenesis.

Our microarray analysis also showed that many genes are regulated by STAT3 in osteoclasts. Indeed, STAT3 may regulate osteoclast resorption function through the lysosome pathway identified by the expression of the gene coding for cathepsin K, Serpinb6b (inhibitor of cathepsin G [38]) and the *ATP6v0d2* gene which encodes the ATPase H+ transporting V0 subunit d 2 (ATP6v0d2). ATP6v0d2 function includes acidification of intracellular organelles [39] and mice with a deficiency in ATP6v0d2 were shown to develop an osteopetrotic phenotype [40]. Additionally, the *hyal1* gene which encodes for lysosomal hyaluronidase [41] was also identified to be upregulated in RAW264.7 cells with knocked down of STAT3. HYAL1 protein levels have been shown to increase during osteoclast differentiation and its extracellular secretion is important for resorption function [42]. We also found an upregulation of NF-kB inhibitor epsilon (NFkBie) when STAT3 was knocked down in RAW264.7 cells, suggesting that STAT3 may regulate the expression of NFkBie, thereby modulating RANK receptor signaling. We also observed changes in the expression of e**arly estrogen-induced gene 1** (*ee1g1*), which encodes a novel RANK signaling component important for osteoclastogenesis. The EE1G1 protein was shown to be recruited to the RANK receptor upon it stimulation, where it facilitates PLCγ2 phosphorylation of NFATc1 induction [43]. Collectively, these data suggest that STAT3 is an important transcriptional factor regulating many genes involved in osteoclast differentiation and function.

Another intriguing pathway that STAT3 may regulate in osteoclasts is the modulation of the oxidative stress within cells. In this context, we observed an upregulation of the gene encoding for the osteoclast-associated receptor (OSCAR) when STAT3 was knocked down. OSCAR is required for osteoclast differentiation but it is involved NFATc1 regulation of oxidative stress-mediated atherogenesis [44]. Likewise, the expression for NHEDC2 is also upregulated in osteoclasts in which STAT3 was knocked down. NHEDC2 is a sodium hydrogen antiporter, which was previously identified as one of the genes downstream of NFATc1 regulation in osteoclasts [45].

More importantly, many of the genes that were identified as possible targets of STAT3 regulation are directly regulated by estrogen. The expression of *lfi202* gene has been shown to be stimulated by IL6 and is directly regulated by STAT3 [46]. The expression of *ifn202* gene is differentially regulated by sex hormones with female hormone 17beta-estradiol significantly increasing its expression while the male hormone dihydrotestosterone decreased expression [47]. SRC expression is upregulated in osteoclasts with knock down of STAT3. SRC protein was shown to be involved in PDGF-mediated tyrosine phosphorylation of both STAT1 and STAT3, thereby activating their transcriptional functions [48]. Osteoclasts from mice with *c-src* gene deletion have lost their bone resorption activity, suggesting that SRC activity may regulate osteoclast function [49]. Furthermore, it appears that SRC is a critical factor in sex hormone-dependent bone mass maintenance [50]. The serotonin transporter (SERT or 5-HTT) is another molecule identified in our screen of osteoclasts when STAT3 is knocked down. Recent studies have shown that SERT-/- mice exhibited a decrease in bone formation and it appears pre osteoclastic cells require serotonin to differentiate into mature osteoclasts [51, 52]. Serotonin modulates the level of estrogen thereby possibly altering the differential effect of either ER-alpha or ER-beta on bone growth and resorption [53]. How the interaction between estrogen and STAT3 affects bone homeostasis merits further investigation.

Our microarray analysis has also identified five genes that appear to be directly regulated by STAT3: CD97, C/EBPa, Gadd45g, HOX-a1, and Sla. Two of particular interest are CD95 and C/EBPa. CD95 is an adhesion G protein-coupled receptor which upon binding to its ligand CD55 activates the Jak2/STAT3 pathway [54]. RANKL was shown to induce CD97 expression and CD97-/- mice exhibited low osteoclast numbers while bone marrow-derived osteoclasts from such mice showed decreased expression of cFos and NFATc1 expression after RANKL stimulation [55]. C/EBPa is an important transcription factor required for osteoclast differentiation [56, 57]. Whether estrogen regulation of CD97 and C/EBPa is STAT3 dependent remains to be determined.

One limitation of this study is that we failed to find the difference in bone formation and resorption between male STAT3 cKO mice and their sex-matchced controls. Because the significant difference in BV/TV between male STAT3 cKO mice and the controls exists at age of 8 weeks, there is a possibility that we could detect a difference in bone formation and/or resorption parameters at a younger age. On the other hand, our data imply a potential interaction between STAT3 and sex hromones because bone formation and resorption differ between male and female STAT3 cKO mice with an increase in age. Another limitation is that RAW264.7 preosteoclast cell line used in these studies was derived from a male mouse which had been transformed by the Abelson murine leukemia virus. In order to control for the variability of working with primary cultures as well as to compare our data with data from other laboratories, we selected to work with this well-known pre-osteoclastic cell line, RAW264.7 cells. However, the male osteoclast cell line could complicate the interpretation and conclusion made especially in regard to its relevance to the role of STAT3 in female osteoclasts.

Collectively, our data suggest that STAT3 functions in regulating trabecular bone development. Our findings also suggest that STAT3 may be linked to estrogen and this may explain the more severe phenotype of a STAT3 knockdown in female bones compared with male bones. STAT3 regulates osteoclast differentiation and activity via cathepsin K. STAT3 signaling may interact with estrogen signaling pathways. Further, our data reported here show the complicated nature of STAT3 signaling pathways in osteoclasts. Finaly, modulation of STAT3 signaling may be targeted to treat bone metabolic diseases, such as Job’s Syndrome and menopause-associated osteoporosis.

## Supporting information

S1 Table Primers used for real-time PCR. (DOCX)

S1 Fig Osteoblast indices measured on Von Kossa stained undecalcified bone sections at the distal femurs. (DOCX)

S1 Data Genes regulated by RANKL and STAT3. (XLSX)
