# Supplementary material for: The loss of STAT3 in mature osteoclasts has detrimental effects on bone structure
Source: PLoS One. 2020 Jul 30;15(7):e0236891. doi: 10.1371/journal.pone.0236891 (PMC7392311; doi:10.1371/journal.pone.0236891)
Supplement: S1 Table — (DOCX) [file pone.0236891.s001.docx]

| **S1 Table. Primers used for real-time PCR** | | |
| --- | --- | --- |
| **Genes** | **Forward Primers** | **Reverse Primers** |
| Stat3 | 5’-TTCAGCGAGAGCAGCAAAG -3' | 5’-AGACAAGTGGAGACACCAG -3' |
| Cathepsin K | 5’-CCCAGACTCCATCGACTATCG-3' | 5’-CTGTACCCTCTGCCATTAGCTGCC-3' |
| Atp6v0d2 | 5’-GACCCTGTGGCACTTTTTGT-3' | 5’-GTGTTTGAGCTTGGGGAGAA-3' |
| Hyal1 | 5’-CCTTCAGTCCTGAGGTTTCCC-3' | 5’-CTCCATACTCCGTCAGGCAC-3' |
| Oscar | 5’-TGATTGGCACAGCAGGAG-3' | 5’-AAGGCACAGGAAGGAAATAGAG-3' |
| Nfkbie | 5’-GGCTGAGGACCTCCTTTCTT-3' | 5’-CCATCCGAGCTTCAGTCAGTA-3' |
| Src | 5’-CCAGGCTGAGGAGTGGTACT-3' | 5’-CAGCTTGCGGATCTTHTAGT-3' |
| Serpinb6b | 5’-GCCAATATAGGTTTTAGGTGATATGGTCC-3' | 5’-GCTATGCAGTTGAGGCTAGCCCTGCATG-3' |
| Mapk11 | 5’-GGCTGATGAGGAGATGACCG-3' | 5’-CAGCTGGTCGATGTAGTCGT-3' |
| Nhedc2 | 5’-GCAGCTGGATCTTTTCTTGG-3' | 5’-TCTTCCAGCTGTTCTCTCC-3' |
| Fam102a (EEIG1) | 5’-CTCAGTATGGCTGTAGAGGG-3' | 5’-ATCTTCTCCACAATGTCGTC-3' |
| Slc6a4 (5-HTT) | 5’-GACCAGTGTGGTGAACTGCATGAC-3' | 5’-GATGATGGCAAAGAATGTGGATGCTG-3' |
| Cd97 | 5’-CCCGGCACTTTCCTGTTACT-3' | 5’-AAATGCACACCACAAGCAGG-3' |
| Cebpa | 5’-ATAAAGCCAAACAGCGCAAC-3' | 5’-CGGTCATTGTCACTGGTCAA-3' |
| Gadd45g | 5’-GGATAACTTGCTGTTCGTGGA-3' | 5’-AAGTTCGTGCAGTGCTTTCC-3' |
| Hoxa1 | 5’-CAGGAAGCAGACCCACCAAG-3' | 5’-CCACGTAGCCGTACTCTCTCCA-3' |
| Sla | 5’-AGATTGGTGCTTCATGATTCG-3' | 5’-GGTGAGTCTCACCTGCTTAG-3' |
| GAPDH | 5’-ACCACAGTCCATGCCATCAC-3' | 5’-TCCACCACCCTGTTGCTGTA-3' |
