# Supplementary material for: The loss of STAT3 in mature osteoclasts has detrimental effects on bone structure
Source: PLoS One. 2020 Jul 30;15(7):e0236891. doi: 10.1371/journal.pone.0236891 (PMC7392311; doi:10.1371/journal.pone.0236891)
Supplement: S1 Fig — (DOCX) [file pone.0236891.s002.docx]

**S1 Fig.1**


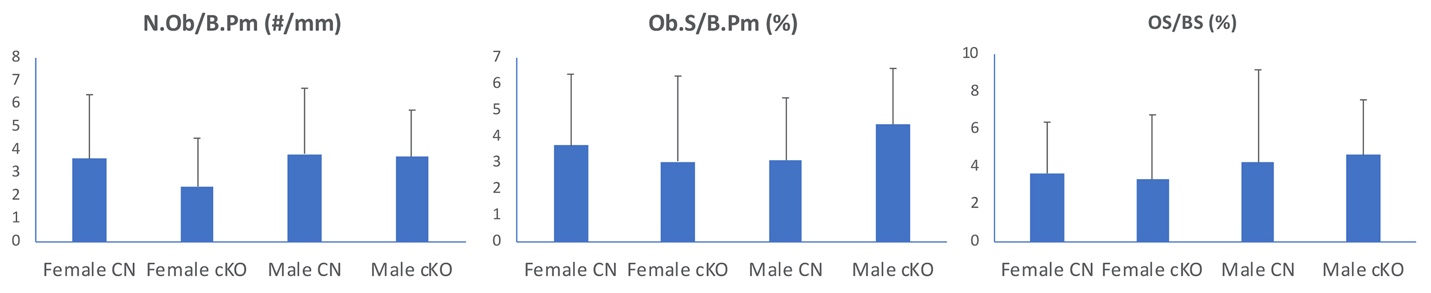


**S1 Fig. 1.** Osteoblast indices measured on Von Kossa stained undecalcified bone sections at the distal femurs. Osteoblast number (N.Ob/B.Pm), osteoblast surface (Ob.S/B.Pm) and osteoid surface (OS/BS) were not significantly different between Stat3 cKO and control groups.
